# Translocation of outer membrane vesicles from enterohemorrhagic *Escherichia coli* O157 across the intestinal epithelial barrier

**DOI:** 10.3389/fmicb.2023.1198945

**Published:** 2023-05-25

**Authors:** Daniel Krsek, Daniel Alejandro Yara, Hana Hrbáčková, Ondřej Daniel, Andrea Mančíková, Stephanie Schüller, Martina Bielaszewska

**Affiliations:** ^1^Centre for Epidemiology and Microbiology, National Institute of Public Health, Prague, Czechia; ^2^Norwich Medical School, University of East Anglia, Norwich, United Kingdom

**Keywords:** enterohemorrhagic *Escherichia coli*, outer membrane vesicles, intestinal epithelial barrier, Caco-2 cells, human colonoids, translocation, hemolytic uremic syndrome

## Abstract

Outer membrane vesicles (OMVs) carrying virulence factors of enterohemorrhagic *Escherichia coli* (EHEC) are assumed to play a role in the pathogenesis of life-threatening hemolytic uremic syndrome (HUS). However, it is unknown if and how OMVs, which are produced in the intestinal lumen, cross the intestinal epithelial barrier (IEB) to reach the renal glomerular endothelium, the major target in HUS. We investigated the ability of EHEC O157 OMVs to translocate across the IEB using a model of polarized Caco-2 cells grown on Transwell inserts and characterized important aspects of this process. Using unlabeled or fluorescently labeled OMVs, tests of the intestinal barrier integrity, inhibitors of endocytosis, cell viability assay, and microscopic techniques, we demonstrated that EHEC O157 OMVs translocated across the IEB. OMV translocation involved both paracellular and transcellular pathways and was significantly increased under simulated inflammatory conditions. In addition, translocation was not dependent on OMV-associated virulence factors and did not affect viability of intestinal epithelial cells. Importantly, translocation of EHEC O157 OMVs was confirmed in human colonoids thereby supporting physiological relevance of OMVs in the pathogenesis of HUS.

## 1. Introduction

Enterohemorrhagic *Escherichia coli* (EHEC) are global causes of diarrhea, hemorrhagic colitis, and hemolytic uremic syndrome (HUS) (Karch et al., 2005). HUS develops as an extraintestinal complication of EHEC diarrhea in 10–15% of infected children (Tarr et al., 2005) and is a leading cause of acute renal failure in childhood (Siegler and Oakes, 2005). HUS is a thrombotic microangiopathy which mainly affects the microvascular endothelium of the renal glomeruli, but also the large intestine and the brain (Richardson et al., 1988). Clinically, it is defined by the triad of microangiopathic hemolytic anemia, thrombocytopenia, and acute renal insufficiency (Tarr et al., 2005). The mortality of EHEC-associated HUS is 3–5% (Siegler and Oakes, 2005) and up to 30% of survivors develop late sequelae such as hypertension, neurological complications, and chronic renal failure (Rosales et al., 2012). EHEC serotypes O157:H7/H^–^ are the major causes of HUS, but several non-O157 serogroups have also been associated with HUS worldwide (Mellmann et al., 2008a; Valilis et al., 2018).

Shiga toxins (Stx), ribosome-inactivating AB_5_ holotoxins, are the major EHEC virulence factors involved in the microvascular endothelial injury that forms the histopathological basis of HUS (Obrig, 2010; Zoja et al., 2010). Stx2a is the most common Stx type associated with HUS (Karch et al., 2005; Mellmann et al., 2008a). Other EHEC toxins which injure microvascular endothelium and may thus contribute to the pathogenesis of HUS are the cytolethal distending toxin V (CdtV) (Bielaszewska et al., 2005) and EHEC hemolysin (EHEC-Hly) (Aldick et al., 2007). Since EHEC infections are non-bacteremic (Bielaszewska and Karch, 2005), it is generally accepted that the microvascular endothelial injury is caused by toxins, mainly by Stx, which are released by EHEC in the intestine, absorbed across the intestinal epithelium into the circulation, and transported to the target organs. There, they injure the endothelium by various mechanisms, which ultimately leads to apoptosis (Bielaszewska and Karch, 2005; Obrig, 2010; Zoja et al., 2010). Notably, this concept of HUS pathogenesis is based on studies using free, purified EHEC toxins (Bielaszewska et al., 2005; Keepers et al., 2006; Aldick et al., 2007; Obrig, 2010).

However, previous studies demonstrated that substantial proportions of EHEC O157 toxins including Stx2a, CdtV, and EHEC-Hly are released from the bacteria not as free, soluble proteins, but in association with outer membrane vesicles (OMVs) (Kolling and Matthews, 1999; Yokoyama et al., 2000; Aldick et al., 2009; Kim et al., 2011; Bielaszewska et al., 2013, 2017). OMVs are nanoparticles (diameter 20–250 nm) ubiquitously produced by Gram-negative bacteria which play multiple roles in interbacterial and bacteria-host communications including disease pathogenesis (Kim et al., 2011; Choi et al., 2015; Schwechheimer and Kuehn, 2015; Jan, 2017; Park et al., 2017; Caruana and Walper, 2020; Haas-Neill and Forsythe, 2020; Rueter and Bielaszewska, 2020; Díaz-Garrido et al., 2021; Villageliu and Samuelson, 2022). EHEC O157 OMVs deliver the associated toxins into human microvascular endothelial cells, where the toxins separate from OMVs, are trafficked to their cellular targets, and cause endothelial injury and ultimately apoptosis (Bielaszewska et al., 2013, 2017). Moreover, EHEC O157 OMV-associated lipopolysaccharide (LPS) and flagellin induce secretion of interleukin 8 (IL-8) from human intestinal epithelial cells (Bielaszewska et al., 2018). This may also contribute to the pathogenesis of HUS where proinflammatory cytokines play important roles (Fitzpatrick et al., 1992; Zoja et al., 2010). OMVs are thus potent EHEC virulence tools that may be involved in the pathogenesis of HUS. Indeed, the ability of EHEC O157 OMVs to cause an HUS-like disease has been demonstrated in a mouse model (Kim et al., 2011).

To reach the microvascular endothelium and cause HUS, OMVs released by EHEC in the intestinal lumen must translocate across the intestinal epithelial barrier (IEB) into the bloodstream. Although OMV transport across the intestinal epithelium has been reported for the intestinal microbiota (Choi et al., 2015; Park et al., 2017; Stentz et al., 2018; Haas-Neill and Forsythe, 2020; Jones et al., 2020; Tulkens et al., 2020; Díaz-Garrido et al., 2021; Hendrix and De Wever, 2022), the ability of EHEC OMVs to cross the IEB has not been investigated. In the mouse study of OMV-mediated HUS (Kim et al., 2011), OMVs were administered intraperitoneally allowing them to circumvent the intestinal barrier.

Here, we investigated the ability of EHEC O157 OMVs to translocate across the IEB using a model of polarized Caco-2 cells grown on Transwell inserts that exhibit characteristics of matured enterocytes including the presence of apical microvilli and functional tight junctions (TJs) (Hidalgo et al., 1989; Ghaffarian and Muro, 2013). We analyzed the routes of OMV translocation, the influence of inflammation, and the roles of OMV-associated virulence factors in this process. Our data demonstrate that EHEC O157 OMVs translocate across the IEB by both paracellular and transcellular pathways, translocation is significantly increased under simulated inflammatory conditions, and independent of OMV virulence factor cargoes. Moreover, translocation of OMVs across human colonoids supports a relevant role of this process in HUS development.

## 2. Materials and methods

### 2.1. Sources, isolation and characterization of EHEC O157 OMVs

Enterohemorrhagic *Escherichia coli* strains 5791/99 (O157:H7), 258/98-1, and 258/98-2 (both O157:H^–^) were isolated from patients with HUS (Friedrich et al., 2006; Mellmann et al., 2008b). Strains 258/98-1 and 258/98-2 originated from sequential stools of the same patient; the initial isolate (258/98-1) carried the *stx*_2*a*_ and *cdt*V genes, whereas the follow-up one (258/98-2) lost the *stx*_2*a*_ gene during infection (Mellmann et al., 2008b) and, subsequently, the *cdt*V cluster during laboratory storage (this study). EHEC O157:H7 strain 85–170 is a derivative of isolate 84–289 which spontaneously lost the ability to produce Stx1 and Stx2 during laboratory storage (Tzipori et al., 1987). OMVs were collected by ultracentrifugation and purified by OptiPrep (Iodixanol) (Sigma-Aldrich) density gradient fractionation as described previously (Bielaszewska et al., 2013, 2021). OMV-associated virulence proteins (Stx2a, CdtV, EHEC-Hly, flagellin) were detected by immunoblotting (Bielaszewska et al., 2017, 2021) and LPS was quantified with the LAL Chromogenic Endotoxin Quantitation Kit (Thermo Fisher Scientific). OMV sizes and counts were determined by nanoparticle tracking analysis with NanoSight LM10 instrument (NanoSight) as described previously (Bauwens et al., 2017b). Protein concentration was measured with the Roti-Nanoquant reagent (Carl Roth). Characteristics of EHEC O157 OMVs are summarized in Supplementary Table 1. A non-pathogenic *E. coli* strain was isolated from stool of a healthy person. OMVs from this strain isolated as described above lacked all EHEC O157 toxins (Stx2a, CdtV, EHEC-Hly), O157 LPS, and H7 flagellin.

### 2.2. Establishment of polarized Caco-2 monolayers

Caco-2 cells (human colorectal adenocarcinoma cell line, ACC 169, German collection of microorganisms and cell cultures, Braunschweig, Germany) were cultured in Eagle’s Minimum Essential Medium (EMEM) with Earle’s salts (Sigma-Aldrich) supplemented with 10% fetal bovine serum (FBS) (Sigma-Aldrich), 2 mM L-glutamine and 1% non-essential amino acids (Lonza). To establish polarized monolayers, cells were seeded into Corning Transwell polyester membrane inserts (6.5 mm diameter, 0.4 μm pores) in 24-well plates (Sigma-Aldrich) at a density of 1.3 × 10^5^ cells/insert and cultured (37°C, 5% CO_2_) for 19–21 days. Growth was monitored by microscopy and polarization was determined by measuring transepithelial electrical resistance (TEER) using the Epithelial Volt/Ohm Meter 3 (EVOM3) with STX2-PLUS electrode (World Precision Instruments). Cells were used after TEER reached ≥350 Ohms × cm^2^ (Ghaffarian and Muro, 2013). All experiments were performed in EMEM without FBS.

### 2.3. Human colonic organoid culture

This study was performed with approval from the University of East Anglia Faculty of Medicine and Health Sciences Research Ethics Subcommittee (Application ETH2122-1185). Samples were collected by the Norwich Research Park Biorepository (REC reference 19/EE/0089). Biopsy samples from macroscopically normal areas of the transverse colon were obtained with informed consent during colonoscopy of a 60-year old male patient. Colonoids were established as described previously (In J. et al., 2016) with the following modifications. Colonic crypts were dissociated by incubation of tissue fragments in 17 mM ethylenediaminetetraacetic acid (EDTA) (Fisher Scientific) in cold chelating solution for 30 min at 4°C. Washed crypt pellets were seeded in Matrigel (Corning) and grown in expansion medium composed of Advanced Dulbecco’s Modified Eagle’s Medium/Ham’s Nutrient Mixture F-12 (Life Technologies) supplemented with 10 mM HEPES (Life Technologies), 2 mM GlutaMAX (Life Technologies), 50% (v/v) Wnt3a-conditioned medium, 20% (v/v) R-spondin 1-conditioned medium, 10% (v/v) Noggin-conditioned medium, 1× B27 supplement (Life Technologies), 10 mM nicotinamide (Sigma-Aldrich), 1 mM N-acetylcysteine (Sigma-Aldrich), 50 ng/ml human epidermal growth factor (Life Technologies), 10 nM [Leu-15] gastrin (AnaSpec), 500 nM A83-01 [3-(6-methylpyridin-2-yl)-N-phenyl-4-quinolin-4-ylpyrazole-1-carbothioamide] (Tocris), 10 μM SB202190 [4-[4-(4-fluorophenyl)-5-pyridin-4-yl-1,3-dihydroimidazol-2-ylidene]cyclohexa-2,5-dien-1-one] (Sigma-Aldrich), 100 μg/ml primocin (Invivogen) and 10 μM Y-27632 [4-[(1R)-1-aminoethyl]-Npyridin-4-ylcyclohexane-1-carboxamide] (Tocris).

For incubation with OMVs, fragmented colonoids were seeded on Transwell inserts (6.5 mm diameter, 0.4 μm pores) coated with human type IV collagen (Sigma-Aldrich) (10 μg/cm^2^) and grown in expansion medium until confluent (7–10 days). Colonoid monolayers were subsequently differentiated by withdrawal of SB202190, Wnt3a and R-spondin 1 for 3–5 days.

### 2.4. Quantification of OMV translocation across polarized Caco-2 monolayers

Outer membrane vesicles were labeled with the fluorescent membrane dye 3,3 dioctadecyloxacarbocyanine perchlorate (DiO) using Vybrant DiO cell-labeling solution (Molecular Probes) as described previously (Bielaszewska et al., 2021). Briefly, 500 μl OMVs (containing ∼ 200 μg OMV protein) were mixed with 4.5 ml PBS and 50 μl Vybrant DiO solution (final DiO concentration of 1%). The mixture was incubated at 37°C for 30 min in the dark with gentle shaking. To remove unbound dye, labeled OMVs were washed twice with 70 ml phosphate-buffered saline (PBS) using ultracentrifugation (235.000 × *g*, 2 h, 4°C), resuspended in 500 μl PBS and stored on ice until use. For translocation experiments, DiO-labeled OMVs were applied into apical compartments (ACs) of Transwell inserts with polarized Caco-2 monolayers (30 μg of OMV protein/insert) and incubated (37°C, 5% CO_2_) for 24 h. Aliquots were collected from basolateral compartments (BCs) after 1, 2, 3, 4, and 24 h of incubation. Fluorescence was measured with a microplate reader (Infinite M200, Tecan) at 485/544 nm (excitation/emission) and expressed as percentage of DiO-OMV fluorescence applied to ACs (defined as 100%).

In the *in vitro* colitis model (Tulkens et al., 2020), Caco-2 monolayers were preincubated for 24 h with 3% (w/v) dextran sulfate sodium (DSS) (Sigma-Aldrich) in cell culture medium applied to ACs. After removing DSS, cells were washed twice with PBS (Lonza) and DiO-labeled OMVs (30 μg/insert) were applied into ACs. Aliquots were collected from BCs after 1, 2, 3, 4, and 24 h of incubation and fluorescence was measured and evaluated as described above. The effect of DSS on TJs was verified by confocal laser scanning microscopy after staining with anti-zonula occludens-1 (ZO-1) or anti-occludin mouse monoclonal antibody and Alexa Fluor 488-conjugated goat anti-mouse IgG (all Invitrogen).

For electron microscopy, unlabeled OMVs (30 μg/insert) or cell culture medium only (negative control) were applied into ACs of polarized Caco-2 monolayers and incubated for 24 h. Medium was then collected from BCs and OMVs were detected by negative staining with 2% (w/v) uranyl acetate (Bielaszewska et al., 2021) using a Hitachi HT7800 electron microscope (Hitachi HighTech).

### 2.5. Monitoring of TEER

Eagle’s Minimum Essential Medium with FBS was removed from polarized Caco-2 monolayers grown on Transwell filters and replaced with EMEM without FBS. The cultures were equilibrated at 37°C and 5% CO_2_ for 30 min and TEER (*t* = 0 h) was measured using the EVOM3 voltohmmeter. OMVs (30 μg/insert) were then applied into ACs and TEER was measured after 1, 2, 3, 4, and 24 h of incubation. 5 mM EDTA (Invitrogen) served as a positive control and cell culture medium as a negative control. TEER of an insert without cells (a background filter resistance) was measured in parallel and subtracted from TEER values of the samples. TEER values at each time point were normalized to the insert area (× 0.3316 cm^2^) and expressed as the percentage of TEER at the time 0 h.

### 2.6. Monitoring of fluorescently labeled dextran flux

Rhodamine B isothiocyanate-dextran (RhB-dextran; MW 70 kDa) (Sigma-Aldrich) at 2 mg/ml was applied into ACs of Transwell inserts with polarized Caco-2 monolayers together with: (i) EHEC O157 OMVs (30 μg/insert); or (ii) 5 mM EDTA (positive control); or (iii) cell culture medium (negative control). Fluorescence was measured in BCs after 1, 2, 3, 4, and 24 h using a microplate reader at 520/590 nm (excitation/emission). At each time point, RhB-dextran flux into BC was expressed as percentage of RhB-dextran fluorescence applied to AC (defined as 100%).

### 2.7. Investigation of transcellular OMV translocation

Polarized Caco-2 monolayers were preincubated (1 h, 37°C) with inhibitors of endocytosis including dynasore (80 μM), chlorpromazine (15 μg/ml), filipin III (10 μg/ml), or amiloride (10 mM) (all Sigma-Aldrich) or left untreated (control). DiO-labeled OMVs (30 μg/insert) were applied into ACs and DiO fluorescence was measured in BCs after 24 h of incubation as described above. Fluorescence in BCs of inhibitor-pretreated monolayers was expressed as percentage of fluorescence in BCs of untreated monolayers (defined as 100%).

### 2.8. Quantification of cell viability

Polarized Caco-2 monolayers were incubated with OMVs (30 μg/insert), 0.1% (v/v) Triton X-100 (Sigma-Aldrich) (positive control) or cell culture medium (negative control) for 4 or 24 h. Medium from ACs was then removed and Transwell filters were placed into an empty 24-well plate. Cell viability was determined using the CyQUANT MTT Cell Proliferation Assay Kit (Invitrogen) according to the manufacturer’s instructions. Absorbance was measured at 570 nm with a plate reader. The assay is based on the conversion of the yellow tetrazolium salt 3-(4,5-dimethylthiazol-2-yl)-2,5-diphenyl tetrazolium bromide (MTT) by dehydrogenases of living cells into blue formazan, the amount of which is proportional to the number of living cells (Mosmann, 1983).

### 2.9. Immunoblot

To compare OMVs from ACs and BCs for the presence of virulence factors, OMVs from each compartment were subjected to immunoblot performed as described previously (Bielaszewska et al., 2021). Briefly, OMVs (15 μg/lane) were separated by sodium dodecylsulfate polyacrylamide gel electrophoresis in a Mini-Protean TGX stain-free gel (BioRad), and transferred to a polyvinylidenfluoride membrane using Trans-Blot Turbo (BioRad). The membrane was blocked with 5% (w/v) skimmed milk, and incubated with antibodies against the outer membrane protein OmpA (OMV marker), Stx2a, CdtV-B, EHEC-Hly or H7 flagellin and alkaline-phosphatase-conjugated goat anti-rabbit IgG. Signals were developed with NBT/BCIP substrate (Roche), visualized with Chemi Doc XRS imager (BioRad), quantified by densitometry (Quantity One, BioRad) and expressed in arbitrary densitometric units (DU).

### 2.10. Vero cell cytotoxicity assay

Vero cells (ACC-33; German collection of microorganisms and cell cultures, Braunschweig, Germany) were cultured in Dulbecco’s modified Eagle medium (DMEM) with 4.5 g/l of glucose and glutamine supplemented with 10% FBS (all Sigma-Aldrich). In the cytotoxicity assay (Kunsmann et al., 2015) semiconfluent Vero cell monolayers grown in 96-well plates (Brand, Life Science) were incubated with 100 μl of two-fold dilutions of OMVs from ACs and BCs for 72 h. Medium with detached cells was then removed, remnant adherent cells were fixed with 2% (v/v) formalin, stained with 0.13% (v/v) crystal violet, washed, and cell detachment was quantified by measuring absorbance (OD_570_) of crystal violet eluted with 50% (v/v) ethanol using a microplate reader. Cytotoxicity titers were expressed as reciprocals of sample dilutions that killed 50% cells.

### 2.11. Confocal laser scanning microscopy

To detect OMV translocation across the model intestinal barrier, OMVs from strain 5791/99 and 85–170 (30 μg/insert) were applied into ACs of Transwell inserts with polarized Caco-2 and human colonoid monolayers, respectively, and incubated for up to 24 h. Monolayers were then washed with PBS, fixed with 4% (w/v) paraformaldehyde, quenched with 0.2 M glycine (pH 7.2), permeabilized with 0.25% (v/v) Triton X-100 and blocked with 5% (w/v) bovine serum albumin (all Sigma-Aldrich). OMVs were stained with rabbit polyclonal anti-*E. coli* O157 LPS antibody (Bielaszewska et al., 2017, 2021) and Cy3-conjugated goat anti-rabbit IgG (Jackson ImmunoResearch) or Alexa Fluor 568-conjugated donkey anti-rabbit IgG (Invitrogen). In colonoids, goblet cells were labeled with mouse anti-MUC2 (Santa Cruz) and Alexa Fluor 647-conjugated donkey anti-mouse IgG (Invitrogen). Filamentous actin and cell nuclei were counterstained with phalloidin-Alexa Fluor 488 (Invitrogen) and 4′,6-diamidino-2-phenylindole (DAPI) (Thermo Scientific), respectively. Monolayers incubated with cell culture medium only served as negative controls.

To visualize OMV translocation pathways, Caco-2 monolayers were incubated with 5791/99 OMVs for 10 min, 15 min, 20 min, 40 min, 1 h, and 4 h and stained for OMVs (anti-*E. coli* O157 LPS antibody and Cy3-conjugated goat anti-rabbit IgG) and ZO-1 (anti-ZO-1 antibody and Alexa Fluor 488-conjugated goat anti-mouse IgG). Nuclei were stained with DAPI. Preparations were mounted in fluorescence mounting medium (Dako) and analyzed with a confocal laser-scanning microscope Leica TCS SP8 with Acousto-Optical Beam Splitter equipped with Diode 405 nm, Argon, and DPSS 561 nm lasers and HCPL APO CS2 63x/1.4 immersion oil objective (Leica Microsystems). Z-stacks at 0.23 μm per slice were acquired using Leica LAS X version 3.5.7.23225 software (Leica Microsystems). 3D images were acquired using Leica LAS X 3D viewer (Leica Microsystems). Images were processed with ImageJ software version 1.53t.

### 2.12. Statistical analysis

Data of two or multiple groups were analyzed with the Student’s *t*-test or One-way Analysis of Variance (ANOVA) with Tukey’s Honest Significant Difference (HSD), respectively, using GraphPad Prism 5 software (version 5.04). *P*-values < 0.05 were considered significant.

## 3. Results

### 3.1. EHEC O157 OMVs translocate across polarized Caco-2 monolayers

**FIGURE 1:**
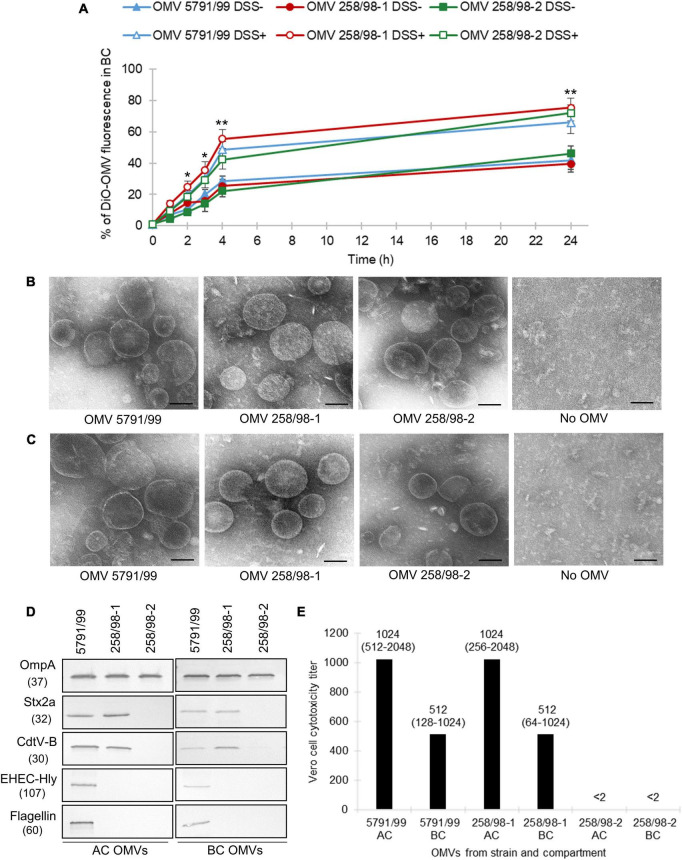
EHEC O157 OMVs translocate across polarized Caco-2 monolayers and translocation is increased under simulated inflammatory conditions and independent of virulence factors. **(A)** Time course of translocation of DiO-labeled OMVs in the absence (DSS-) and presence (DSS+) of dextran sulfate sodium pretreatment. DiO-labeled OMVs 5791/99 (carrying Stx2a, CdtV, EHEC-Hly, and H7 flagellin), 258/98-1 (carrying Stx2a and CdtV), and 258/98-2 (lacking all these virulence factors) were applied into apical compartments (ACs) of Transwell inserts with polarized Caco-2 monolayers pretreated with 3% DSS or left untreated. DiO fluorescence in basolateral compartments (BCs) was measured after indicated time periods and expressed as percentage of the original OMV inoculum. Data are means ± standard deviations from three independent experiments. *p < 0.05 or **p < 0.01 for DiO-OMV translocation after DSS pretreatment compared to untreated controls (applies to DiO-OMVs from all three strains) (Student’s *t* test). **(B,C)** Electron microscopy (uranyl acetate staining) of EHEC O157 OMVs applied to ACs of Transwell-grown polarized Caco-2 monolayers **(B)** and isolated from BCs after 24 h **(C)**. No OMV, control filters where cell culture medium only was applied. Scale bars are 100 nm. Images are representative of two independent experiments. **(D)** Presence of virulence factors in EHEC O157 OMVs applied to ACs (AC OMVs) and isolated from BCs (BC OMVs) as detected by immunoblot. OmpA is an OMV marker. Molecular weights (in kDa) are given below the protein designations. Images are representative of two independent experiments. **(E)** Vero cell cytotoxicity titers of OMVs from ACs and BCs; titers are shown as medians (ranges) from four independent experiments; <2, no cytotoxicity was observed with OMV dilution 1:2.

To determine if EHEC O157 OMVs are capable of translocating across the IEB, DiO-labeled OMVs from strains 5791/99 (carrying Stx2a, CdtV, EHEC-Hly, and H7 flagellin), 258/98-1 (carrying Stx2a and CdtV), and 258/98-2 (lacking all the virulence factors) (Figure 1D) were applied into ACs of Transwell inserts with polarized Caco-2 monolayers and fluorescence was measured in BCs at different time points. The fluorescence in BCs increased in a time-dependent manner and reached 40–46% of that applied into ACs after 24 h (Figure 1A). This indicated that DiO-labeled EHEC O157 OMVs translocated across the IEB. Notably, the extent and kinetics of translocation did not differ significantly in OMVs with different combinations of virulence factors (Figure 1A) suggesting that OMV translocation is independent of OMV-associated toxins (Stx2a, CdtV, and EHEC-Hly) and H7 flagellin. This was further supported by the observation that DiO-labeled OMVs from a non-pathogenic *E. coli* strain (lacking all EHEC toxins, H7 flagellin, and O157 LPS) used as a control displayed similar extent and kinetics of translocation (Supplementary Figure 1) as OMVs from EHEC O157 strains.

To confirm the OMV translocation process, polarized Caco-2 cells were incubated with unlabeled EHEC O157 OMVs (Figure 1B), and media from BCs were examined by electron microscopy after 24 h. OMVs from all strains were detected in BCs (Figure 1C) confirming their translocation across the model IEB. No OMVs were detected in BCs of control inserts where cell culture medium without OMVs was applied into ACs.

To find out if EHEC O157 OMVs underwent changes during translocation, we compared the sizes and virulence factor contents in OMVs applied into ACs and isolated from BCs. OMVs from ACs and BCs did not differ in their sizes as determined by electron microscopy (Figures 1B, C). However, OMVs from BCs contained lower amounts of virulence factors (Stx2a, CdtV, EHEC-Hly, and flagellin) than OMVs from ACs as detected by immunoblot (Figure 1D and Supplementary Figure 2). This suggested that virulence factors separated from a subset of OMVs during their translocation across polarized Caco-2 monolayers. Accordingly, the cytotoxicity of OMVs from BCs toward Vero cells, which are highly sensitive to OMV-associated Stx2a (Kunsmann et al., 2015), was approximately half of that of OMVs applied to ACs (Figure 1E).

### 3.2. OMV translocation is increased by simulated intestinal inflammation

Enterohemorrhagic *Escherichia coli* infection in patients and animal models is accompanied by severe inflammation of the colonic mucosa which compromises intestinal barrier integrity (Griffin et al., 1990; Kelly et al., 1990; Roxas et al., 2010; Schüller, 2011; Ugalde-Silva et al., 2016). To assess if inflammation facilitates OMV translocation across the IEB, we used an *in vitro* colitis model. DiO-labeled OMVs were applied into ACs of polarized Caco-2 monolayers pretreated with 3% DSS which compromises intestinal TJs (Supplementary Figure 3) and induces colitis (Poritz et al., 2007; Wang et al., 2019; Tulkens et al., 2020); fluorescence was measured in BCs. As shown in Figure 1A, DSS pretreatment significantly increased translocation of OMVs from all EHEC O157 strains between 2 and 24 h of incubation compared to untreated monolayers (*p* < 0.05 or *p* < 0.01). This demonstrated that simulated inflammation facilitated OMV translocation across the IEB.

### 3.3. EHEC O157 OMVs translocate via both paracellular and transcellular pathways

To elucidate how EHEC O157 OMVs translocate across the IEB, we addressed two major pathways reported for nanoparticles including OMVs (Ghaffarian and Muro, 2013; Stentz et al., 2018; Jones et al., 2020; Tulkens et al., 2020; Díaz-Garrido et al., 2021): (i) the paracellular pathway via disruption of TJs that interlock adjacent intestinal epithelial cells (Aijaz et al., 2006), and (ii) the transcellular pathway where nanoparticles are internalized via endocytosis, trafficked through the cell body and released at the basolateral cell surface. We used both functional and microscopic approaches to gain insights into the OMV translocation pathways.

#### 3.3.1. Paracellular translocation of EHEC O157 OMVs

As a result of TJ disruption, paracellular translocation is associated with impaired integrity and increased permeability of the IEB. We therefore monitored the effects of EHEC O157 OMVs on the TEER and flux of fluorescently labeled dextran across polarized Caco-2 monolayers as markers of OMV paracellular translocation.

**FIGURE 2:**
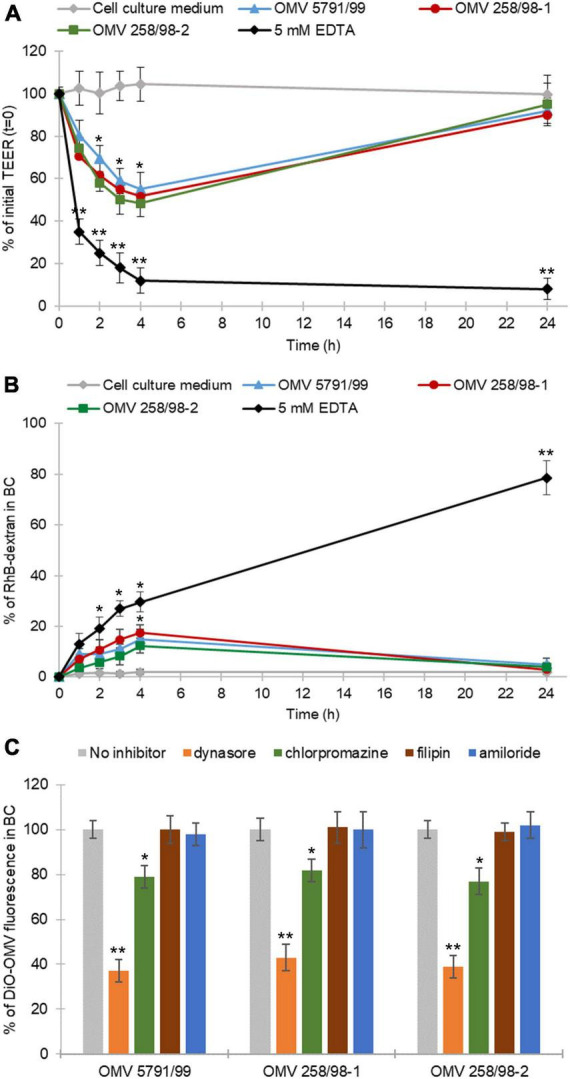
EHEC O157 OMVs translocate via both paracellular and transcellular pathways. **(A)** EHEC O157 OMVs transiently decrease the transepithelial electrical resistance (TEER) and **(B)** transiently increase Rhodamine B isothiocyanate-dextran (RhB-dextran) flux across polarized Caco-2 monolayers. Cells incubated with 5 mM EDTA and cell culture medium alone served as a positive and negative control, respectively. Data are means ± standard deviations from three independent experiments. *p < 0.05 and **p < 0.01 compared to cell culture medium (one-way ANOVA with Tukey’s HSD). **(C)** Translocation of DiO-labeled EHEC O157 OMVs is reduced by inhibitors of dynamin-dependent and clathrin-mediated endocytosis. DiO-labeled OMVs were applied to polarized Caco-2 monolayers pretreated with indicated inhibitors of endocytosis or left untreated. DiO-OMV fluorescence in BCs after 24 h was expressed as percentage of fluorescence of untreated monolayers. Data are means ± standard deviations from three independent experiments. **p < 0.01 or *p < 0.05 for dynasore- or chlorpromazine-pretreated monolayers, respectively, compared to untreated monolayers (one-way ANOVA with Tukey’s HSD).

Outer membrane vesicles from strains 5791/99, 258/98-1, and 258/98-2 caused a transient TEER decrease within 4 h of incubation with a subsequent recovery after 24 h (Figure 2A). No TEER changes were observed in monolayers treated with cell culture medium only, whereas a progressive TEER decrease was caused by 5 mM EDTA (Figure 2A) which depletes calcium and magnesium from intercellular junctions and thus disrupts monolayer integrity (Hurley et al., 2016; Wang et al., 2016).

In addition, all EHEC O157 OMV preparations caused a moderate increase of transepithelial RhB-dextran flux within 4 h of incubation followed by a decrease to the initial (time 0 h) level after 24 h (Figure 2B). Treatment with 5 mM EDTA (positive control) elicited a continuous increase in RhB-dextran flux during 24 h, whereas no dextran flux was observed through monolayers exposed to cell culture medium only (Figure 2B). Notably, there were no significant differences in the effects of OMVs with different sets of virulence factors on TEER (Figure 2A) or dextran flux (Figure 2B). Altogether, these experiments demonstrated that EHEC O157 OMVs caused a transient reduction of IEB integrity indicating paracellular translocation.

To gain insight into the mechanism underlying the transient decrease of IEB integrity, we determined effects of EHEC O157 OMVs on the viability of Caco-2 cells and morphology of the TJ protein ZO-1 after 4 h of incubation, the time point of the lowest TEER (Figure 2A) and the highest RhB-dextran flux (Figure 2B). We found no changes in the viability of Caco-2 monolayers exposed to EHEC O157 OMVs compared to control cells exposed to cell culture medium using the MTT assay (Supplementary Figure 4A). In contrast, confocal microscopy demonstrated a weak and irregular staining of ZO-1 after 4 h of incubation with OMVs compared to intensively stained and intact ZO-1 in control cells (Supplementary Figure 4B). This indicated that the transient decrease in polarized Caco-2 monolayer integrity caused by EHEC O157 OMVs was due to transient disruption of TJs.

#### 3.3.2. Transcellular translocation of EHEC O157 OMVs

The prerequisite for transcellular translocation of OMVs is their internalization by the target cells. We have previously demonstrated that EHEC O157 OMVs are internalized by Caco-2 cells via dynamin-dependent and partially clathrin-mediated endocytosis (Bielaszewska et al., 2017) which is inhibited by dynasore and chlorpromazine, respectively (Wang et al., 1993; Macia et al., 2006). We therefore hypothesized that the inhibition or reduction of OMV translocation by these inhibitors would indicate the involvement of the transcellular pathway in the translocation process. To test this hypothesis, we determined effects of various inhibitors of endocytosis on translocation of DiO-labeled EHEC O157 OMVs. As demonstrated in Figure 2C, pretreatment of polarized Caco-2 monolayers with dynasore or chlorpromazine significantly reduced translocation of DiO-labeled OMVs compared to untreated monolayers (*p* < 0.01 or *p* < 0.05, respectively). In contrast, filipin and amiloride, inhibitors of caveolae-mediated endocytosis and macropinocytosis, respectively (Orlandi and Fishman, 1998; Wadia et al., 2004), which are not involved in endocytosis of EHEC O157 OMVs by Caco-2 cells (Bielaszewska et al., 2017), did not reduce translocation of DiO-labeled OMVs (Figure 2C) confirming the specificity of the dynasore- and chlorpromazine-mediated inhibition. Based on these experiments we conclude that in addition to paracellular transport, the transcellular pathway also plays an important role in the translocation of EHEC O157 OMVs across polarized Caco-2 monolayers.

#### 3.3.3. Confocal laser scanning microscopy analysis of EHEC O157 OMV translocation

**FIGURE 3:**
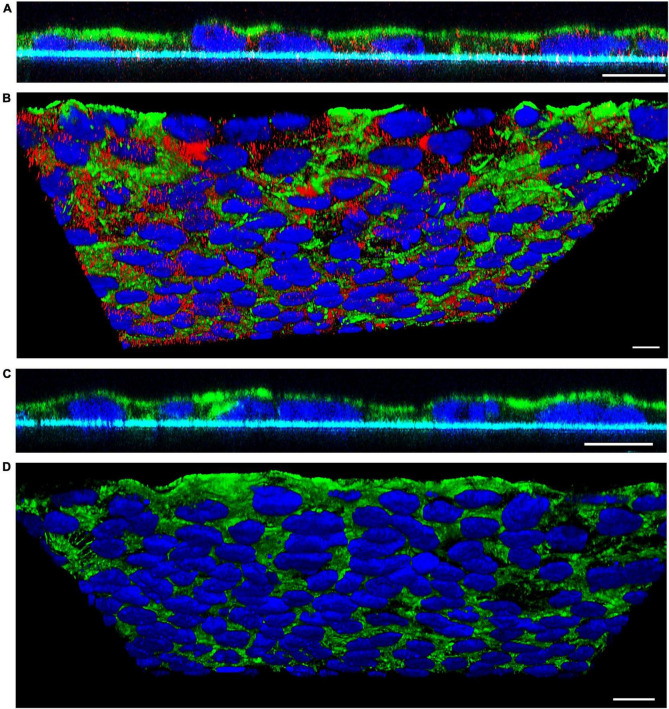
Confocal microscopy analysis of EHEC O157 OMV translocation. Polarized Caco-2 monolayers were incubated for 24 h with OMVs from EHEC O157:H7 strain 5791/99 **(A,B)** or with cell culture medium only **(C,D)**. OMVs (red) were stained with anti-*E. coli* O157 LPS antibody and Cy3-conjugated goat anti-rabbit IgG, actin (green) with phalloidin-Alexa Fluor 488, and nuclei (blue) with DAPI. Preparations were analyzed with a confocal laser-scanning microscope Leica TCS SP8 with a 63x/1.4 immersion oil objective. XZ line scans **(A,C)** were acquired with Leica LAS X software, and 3D images **(B,D)** with Leica LAS X 3D viewer. The blue line below the cells in panels **(A,C)** depicts the Transwell filter. Scale bars are 10 μm. Images are representative of three independent experiments.

To visualize translocation of EHEC O157 OMVs, we performed confocal microscopy of polarized Caco-2 monolayers which had been incubated with OMVs from strain 5791/99 for 24 h and subsequently stained for OMVs and actin. Although rare OMVs still remained on the cell surface, most OMVs were located within monolayers indicating movement through the epithelial barrier (Figure 3A). Numerous OMVs were found in close proximity to the basal membrane or below (Figures 3A, B) demonstrating complete passage across the epithelial barrier. No OMVs were detected in control monolayers exposed to cell culture medium only (Figures 3C, D).

**FIGURE 4:**
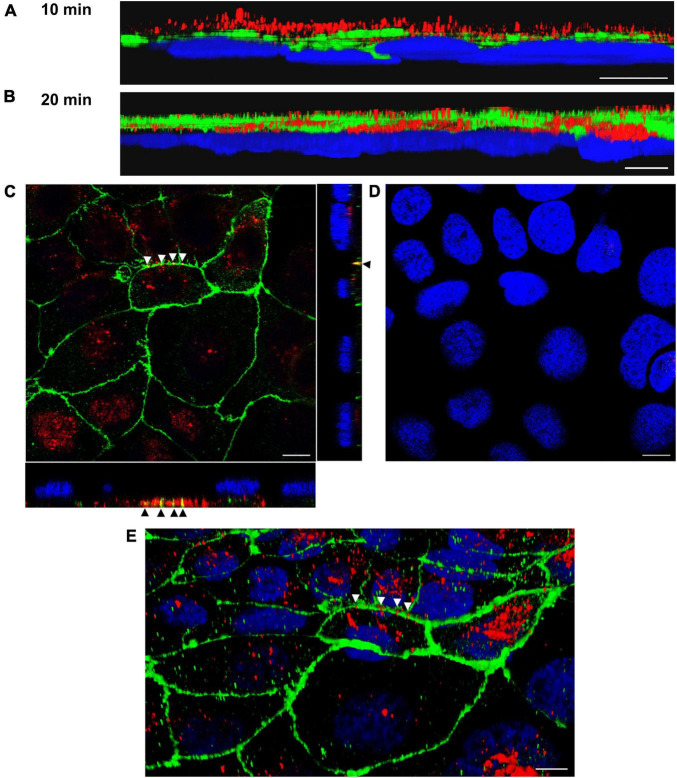
Confocal microscopy analysis of OMV translocation pathways, time points 10 min and 20 min. Polarized Caco-2 monolayers were incubated with OMVs from EHEC O157 strain 5791/99 for 10 min **(A)** or 20 min **(B–E)** and stained for OMVs (anti-*E. coli* O157 LPS antibody and Cy3-conjugated goat anti-rabbit IgG), ZO-1 (anti-ZO-1 antibody and Alexa Fluor 488-conjugated goat anti-mouse IgG), and nuclei (DAPI). Preparations were analyzed with a confocal laser-scanning microscope Leica TCS SP8 with a 63x/1.4 immersion oil objective. 3D images were acquired with Leica LAS X 3D viewer. **(A,B)** Side views of 3D images demonstrating localization of OMVs (red) above ZO-1 (green) after 10 min **(A)** and within and below ZO-1 after 20 min **(B)**. **(C)** Localization of OMVs between cells in colocalization with ZO-1 (yellow signals depicted by arrow heads) and inside cells after 20 min. The main panel shows merged XY images and the side panels orthogonal XZ and ZY projections. **(D)** XY image of the region shown in panel **(C)** at the level of nuclei. **(E)** 3D image (an oblique view) of the region shown in panel **(C)**. Arrow heads depict OMVs (red) crossing ZO-1 (green). Scale bars are 10 μm. Images are representative of three independent experiments.

**FIGURE 5:**
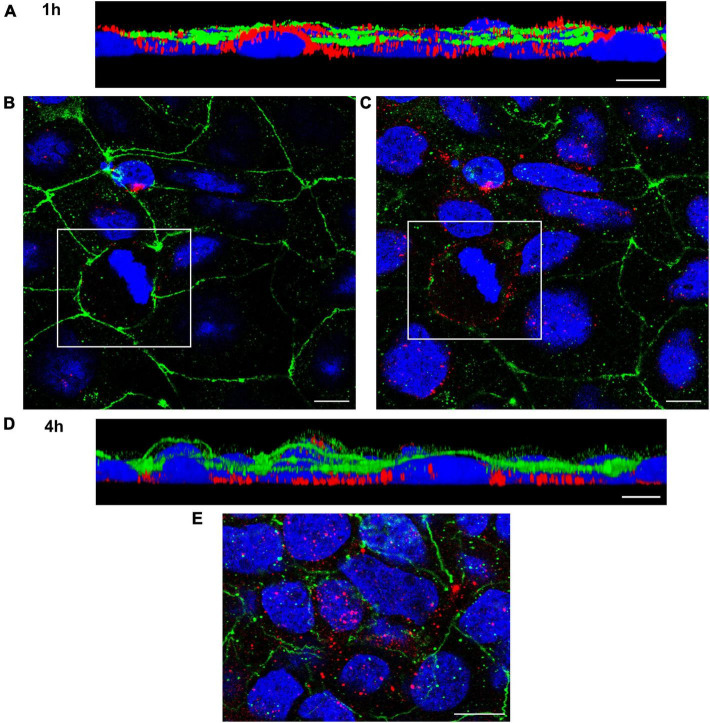
Confocal microscopy analysis of OMV translocation pathways, time points 1 and 4 h. Polarized Caco-2 monolayers were incubated with OMVs from EHEC O157:H7 strain 5791/99 for 1 h **(A–C)** or 4 h **(D,E)** and stained for OMVs (anti-*E. coli* O157 LPS antibody and Cy3-conjugated goat anti-rabbit IgG), ZO-1 (anti-ZO-1 antibody and Alexa Fluor 488-conjugated goat anti-mouse IgG), and nuclei (DAPI). Preparations were analyzed and 3D images were acquired as described in legend to Figure 4. **(A,D)** Side views of 3D images demonstrating localization of OMVs (red) below ZO-1 (green) and at the level of nuclei (blue) after 1 h **(A)** and in the basal cell regions after 4 h **(D)**. **(B,C)** Merged XY images of scans taken at the level of ZO-1 **(B)** and below ZO-1 **(C)**; presence of intercellular ring-like OMV patterns (red) below ZO-1 indicates OMV paracellular translocation [the corresponding areas in panels **(B,C)** are depicted by frames]. **(E)** Localization of OMVs (red) inside cells after 4 h demonstrating transcellular translocation. Scale bars are 10 μm. Images are representative of three independent experiments.

To confirm OMV translocation via paracellular and transcellular pathways, Caco-2 monolayers incubated with EHEC O157 OMVs for 10 min to 4 h were stained for OMVs and the TJ protein ZO-1 and localization of OMVs (inside *versus* between cells) was evaluated. This approach also allowed us to monitor the dynamics of the translocation process. After 10 min of incubation, most OMVs were located on the cell surface above ZO-1 (Figure 4A) suggesting that translocation had not yet started. After 15 to 20 min, OMVs reached the focal level of ZO-1 (Figure 4B and Supplementary Figure 5A). Notably, subsets of OMVs colocalized with ZO-1 (Figures 4C, E and Supplementary Figure 5B) indicating paracellular translocation through TJs. At later time intervals (40 min to 4 h), most of the paracellularly translocated OMVs had passed TJs and could be detected as intercellular ring-like patterns below ZO-1 (Figures 5B, C). Notably, OMVs were also localized inside cells at each time point (Figures 4C, E, 5C, E, and Supplementary Figures 5B, E) indicating transcellular translocation. Taken together, confocal microscopy analysis demonstrated both paracellular and transcellular EHEC O157 OMV translocation across polarized Caco-2 monolayers, thereby confirming results of the functional tests (Figures 2A–C). Moreover, analysis of the translocation dynamics allowed to trace OMV movement from the cell surface through ZO-1 into supranuclear and subnuclear cell regions (Figures 4A, B, 5A, D, and Supplementary Figures 5A, D) providing more details on their transepithelial transport.

### 3.4. EHEC O157 OMV translocation does not affect viability of polarized Caco-2 cells

**FIGURE 6:**
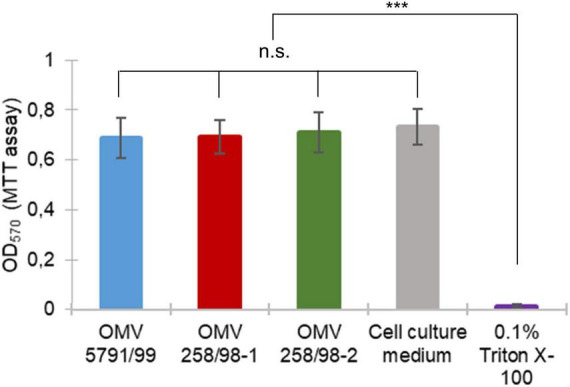
EHEC O157 OMV translocation does not affect viability of polarized Caco-2 cells. Caco-2 monolayers were incubated with EHEC O157 OMVs or controls for 24 h. Cell viability was determined by MTT assay and absorbance at 570 nm (OD_570_). Data are means ± standard deviations from three independent experiments. n.s., not significant; ***p < 0.001 for monolayers incubated with 0.1% Triton X-100 (positive control) compared to monolayers incubated with OMVs or cell culture medium (negative control) (one-way ANOVA with Tukey’s HSD).

To determine whether EHEC O157 OMV translocation affected cell viability, Caco-2 monolayers incubated with EHEC O157 OMVs for 24 h were evaluated by MTT assay. This demonstrated that monolayers exposed to EHEC O157 OMVs retained their viability as indicated by conversion of MTT into formazan (Figure 6). While OD_570_ values of OMV-infected monolayers did not differ significantly from untreated control, Caco-2 cells exposed to 0.1% Triton X-100 as a positive control showed a complete loss of viability (Figure 6). Taken together, these data demonstrated that EHEC O157 OMV translocation did not affect viability of polarized Caco-2 monolayers.

### 3.5. EHEC O157 OMVs translocate across human colonoids

**FIGURE 7:**
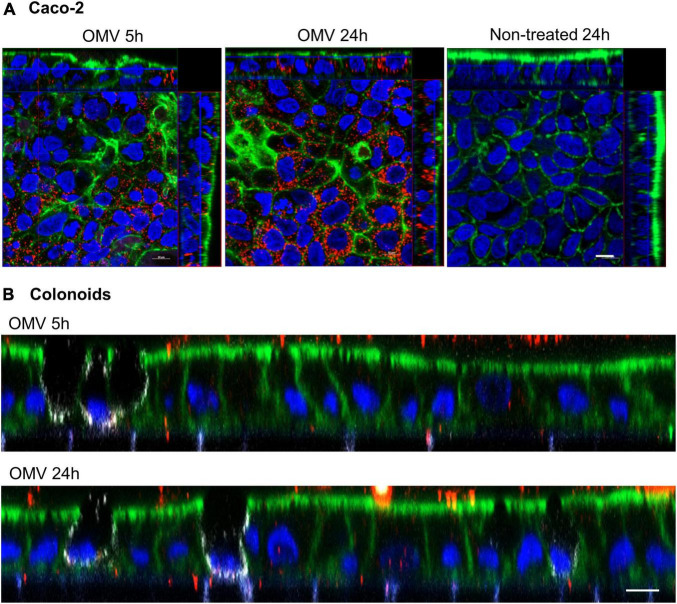
EHEC O157 OMVs translocate across human colonoids. Polarized Caco-2 **(A)** or human colonoid monolayers **(B)** were incubated with OMVs from EHEC O157:H7 strain 85-170 or cell culture medium only (non-treated) for 5 h or 24 h. OMVs (red) were stained with anti-*E. coli* O157 LPS antibody and Alexa Fluor 568-conjugated donkey anti-rabbit IgG, goblet cells (white, colonoids only) with anti-MUC2 and Alexa Fluor 647-conjugated donkey anti-mouse IgG, actin (green) with Alexa Fluor 488-phalloidin and nuclei (blue) with DAPI. Shown are orthogonal projections of Z-stacks for Caco-2 cells **(A)** and XZ line scans for colonoids **(B)**. Scale bars are 10 μm. Images are representative of three **(A)** and two **(B)** independent experiments.

To confirm the relevance of EHEC OMV translocation in a model system more closely related to the *in vivo* situation, human intestinal organoids derived from adult stem cells of human colonic tissue (colonoids) were used. In contrast to enterocyte-derived Caco-2 cells, colonic organoids also include other intestinal epithelial cell types (e.g., mucus-secreting goblet cells and hormone-producing neuroendocrine cells) and have been successfully employed in elucidating host-pathogen interactions (In J. G. et al., 2016). Similar to their transport across polarized Caco-2 cells (Figure 7A), OMVs from EHEC O157 strain 85–170 were internalized by human colonoids after 5 h and released from the basolateral cell surface after 24 h of incubation (Figure 7B). Thus, EHEC O157 OMVs translocate across human colonoids which further supports their ability to translocate across human intestinal mucosa during EHEC infection.

## 4. Discussion

Similar to other Gram-negative bacteria, EHEC O157:H7/H^–^, the most common causes of HUS in children (Karch et al., 2005; Mellmann et al., 2008a) produce OMVs that contain the major virulence factors of these pathogens and are assumed to play a role in the pathogenesis of HUS (Kim et al., 2011; Bielaszewska et al., 2017, 2018; Rueter and Bielaszewska, 2020). Using a model of polarized Caco-2 cells cultured in Transwell inserts, we demonstrate that EHEC O157 OMVs translocate across the IEB. Importantly, the relevance of this process was confirmed in human colonoids which more closely resemble a native intestinal epithelium as they contain all major cell types including mucus-secreting goblet cells (In J. G. et al., 2016). Notably, less OMVs were observed in colonoids compared with Caco-2 cells which might be due to lower endocytic activity or the presence of a mucus barrier. Internalization and transmigration across mouse intestinal organoids has previously been demonstrated for OMVs from the gut commensal *Bacteroides thetaiotaomicron* (Jones et al., 2020) but no OMV studies using human tissue have been performed to our knowledge. Translocation through the intestinal epithelium is crucial to the pathogenetic involvement of OMVs in HUS since it enables OMVs produced by EHEC bacteria in the intestine to reach the target cells, i.e., the microvascular endothelium in the renal glomeruli (Richardson et al., 1988). Here, we characterized various aspects of EHEC O157 OMV epithelial translocation, some of which may be relevant to OMV involvement in HUS development.

First, we demonstrated that EHEC O157 OMV translocation across polarized Caco-2 cells involves both paracellular and transcellular pathways. This was supported by: (i) OMV-mediated transient impairment of monolayer integrity (Figures 2A, B); and (ii) significant reduction of OMV translocation by inhibitors of dynamin- and clathrin-dependent endocytosis (Figure 2C), which is involved in EHEC O157 OMV uptake by Caco-2 cells (Bielaszewska et al., 2017). The parallel involvement of both paracellular and transcellular translocation pathways of EHEC O157 OMVs was confirmed by confocal microscopy (Figures 4, 5, and Supplementary Figure 5). This is in agreement with studies investigating translocation of OMVs from other intestinal bacterial species. In particular, OMVs from *B. thetaiotaomicron* caused, like EHEC O157 OMVs (Figure 2A), a transient impairment of TEER in polarized Caco-2 monolayers and their paracellular translocation was shown microscopically in murine ceacal organoid monolayers (Jones et al., 2020). Similar to our results, parallel transcellular translocation of *B. thetaiotaomicron* OMVs was supported by significant reduction of OMV uptake by inhibitors of endocytosis (Jones et al., 2020). Paracellular translocation was also shown for membrane vesicles from the human intestinal microbiota which were detected in the plasma of patients with intestinal barrier disorders (Tulkens et al., 2020), whereas transcellular translocation through body barriers was reported for *Helicobacter pylori* OMVs in a mouse model of Alzheimer disease (Xie et al., 2023). It is evident that OMVs from different or even the same species use different translocation pathways. This might be caused by differences in OMV size and molecular cargo (Aldick et al., 2009; Bielaszewska et al., 2017; Jones et al., 2020; Tulkens et al., 2020) which can influence their route of epithelial barrier translocation (Díaz-Garrido et al., 2021). Moreover, the specific conditions under which OMVs are produced, such as the intestinal milieu or the presence of antibiotics affect the secretion and characteristics of OMVs (Orench-Rivera and Kuehn, 2016; Bauwens et al., 2017a,b; Devos et al., 2017; Yara, 2020) and might thus influence their mode of translocation.

Second, translocation of EHEC O157 OMVs was significantly increased by pretreatment of the model barrier with DSS (Figure 1A) which compromises TJs and thus mimics colitis (Poritz et al., 2007; Wang et al., 2019; Tulkens et al., 2020). This is of particular relevance to the *in vivo* situation as EHEC bacteria infecting the colonic mucosa cause disruption and redistribution of TJs which results in decreased IEB function (Roxas et al., 2010; Schüller, 2011; Ugalde-Silva et al., 2016). Moreover, EHEC infection triggers recruitment of polymorphonuclear leukocytes (neutrophils) to the infected mucosa (Hurley et al., 2001), which also contributes to TJ disruption (Hurley et al., 2001; Schüller, 2011). Similar to other intestinal inflammatory disorders (Tulkens et al., 2020), colonic inflammation during EHEC O157 infection (Griffin et al., 1990; Kelly et al., 1990) and the resulting intestinal barrier dysfunction (Roxas et al., 2010; Schüller, 2011) likely facilitate OMV translocation from the intestinal lumen to the bloodstream thereby increasing OMV access and interaction with microvascular endothelial cells. Since OMVs contain a substantial proportion (more than 50%) of Stx2a produced by EHEC O157 strains (Bielaszewska et al., 2017), this may increase the risk of HUS development. Notably, EHEC OMVs can substantially contribute to colonic inflammation by inducing secretion of the proinflammatory cytokine IL-8, a potent neutrophil chemoattractant (Fitzpatrick et al., 1992; Hurley et al., 2001), from human intestinal epithelial cells (Kunsmann et al., 2015; Bielaszewska et al., 2018). Through their proinflammatory potential, EHEC OMVs may thus facilitate their own translocation from the intestine to the bloodstream. In addition, the amount of OMVs crossing the intestinal barrier during EHEC infection is further enhanced due to upregulation of OMV production by the stress conditions encountered by EHEC in the human gastrointestinal tract (Bauwens et al., 2017b; Yara, 2020). Although the presence of EHEC OMVs in the circulation of HUS patients has not been investigated, membrane vesicles from intestinal microbiota have been detected in the plasma of patients with intestinal barrier disorders such as inflammatory bowel disease, therapy-induced intestinal mucositis or human immunodeficiency virus infection (Tulkens et al., 2020; Hendrix and De Wever, 2022). These findings support our *in vitro* results and suggest that intestinal inflammation also plays an important role in epithelial OMV translocation during EHEC infection.

Third, in our model, EHEC O157 OMV translocation was independent of Stx and other virulence factors as demonstrated by the similar extent and kinetics of translocation of OMVs with different virulence factor cargoes (Figure 1A). This is supported by our previous study which demonstrated that internalization of EHEC OMVs by intestinal epithelial cells, which precedes transcellular translocation, did not require the presence of the Stx receptor globotriaosylceramide (Gb3) (Bielaszewska et al., 2017). Moreover, our data are in agreement with the observation by Roxas et al. (2010) that an Stx-negative EHEC derivative caused redistribution of TJ proteins and impairment of intestinal barrier function in a mouse model suggesting that non-Stx factors are implicated in the intestinal barrier dysfunction caused by EHEC bacteria. The components of OMVs (and other bacterial vesicles) and the host mechanisms involved in intestinal vesicle translocation are presently unknown. Unraveling these mechanisms in future studies is necessary to expand our knowledge on the spread of gut microbe-derived vesicles from the intestine to distal organs (Choi et al., 2015; Park et al., 2017; Jones et al., 2020; Bittel et al., 2021) and thereby to elucidate the vesicle impact on bacteria-host interactions and their emerging significance in pathogenesis of human diseases (Choi et al., 2015; Park et al., 2017; Hendrix and De Wever, 2022; Xie et al., 2023).

Fourth, our finding of reduced virulence factor contents in OMVs isolated from BCs compared to OMVs applied to ACs suggests that a subset of OMVs underwent a processing during translocation resulting in the loss of virulence factor cargoes. This is in accordance with our previous observations that Stx2a, CdtV-B, and EHEC-Hly separate from OMVs following OMV internalization by the target cells in order to execute their biological functions (Bielaszewska et al., 2013, 2017). Since OMV internalization precedes the transcellular translocation, we hypothesize that the reduction of OMV virulence factor contents results from separation of virulence factors from transcellularly translocated OMVs whereas OMVs translocated paracellularly retain their virulence factors. Importantly, despite the reduced virulence factor contents following translocation, OMVs from BCs demonstrated a considerable cytotoxicity to Vero cells (Figure 1E), a highly Stx-sensitive cell line (Konowalchuk et al., 1977) used as a gold standard for testing Stx cytotoxicity. This supports the cytotoxic potential of EHEC O157 OMVs translocated from the intestine during EHEC infection against their *in vivo* targets, the human glomerular endothelial cells, which are, like Vero cells (Kunsmann et al., 2015), highly sensitive to EHEC O157 OMV-mediated toxic injury (Bielaszewska et al., 2017). In contrast, Caco-2 cells, though they express the Stx receptor Gb3 (Kouzel et al., 2017) and are sensitive to free, purified Stx2a after 24 h of exposure (Schüller et al., 2004), display low (almost 400-fold less than Vero cells) and delayed (after 48 h and longer) sensitivity to OMV-associated Stx2a (Kunsmann et al., 2015; Bielaszewska et al., 2017). This enables EHEC O157 OMV translocation across polarized Caco-2 monolayers without affecting viability of the model IEB.

## 5. Conclusion

We demonstrated that OMVs from EHEC O157 translocate across model intestinal epithelial barriers including polarized Caco-2 cells and human colonoids. The epithelial translocation enables OMVs produced by EHEC bacteria in the gut to reach the glomerular microvascular endothelium and is thus crucial for OMV involvement in HUS, a severe complication of EHEC infection. Our study supports the role of OMVs as EHEC O157 virulence tools and contributes to an increasing body of evidence that bacterial OMVs play important roles in pathogenesis of human diseases.

## Data availability statement

The original contributions presented in this study are included in the article/Supplementary material, further inquiries can be directed to the corresponding author.

## Ethics statement

The studies involving human participants were reviewed and approved by the Research Ethics Subcommittee of the Faculty of Medicine and Health Sciences, University of East Anglia (Application ETH2122-1185). Samples for preparing human colonoids were collected by the Norwich Research Park Biorepository (REC reference 19/EE/0089). Biopsy samples from the transverse colon were obtained with informed consent during colonoscopy of a 60-year old male patient. The patients/participants provided their written informed consent to participate in this study.

## Author contributions

MB and SS conceived and designed the experiments, supervised the work, and wrote the manuscript. DK, DY, MB, SS, HH, OD, and AM carried out the experiments. MB, SS, DK, and DY performed the data analysis and interpretation. DK, OD, and DY prepared the figures. All authors contributed to the article and approved the submitted version.
