# Supplementary material for: Translocation of outer membrane vesicles from enterohemorrhagic Escherichia coli O157 across the intestinal epithelial barrier
Source: Front Microbiol. 2023 May 25;14:1198945. doi: 10.3389/fmicb.2023.1198945 (PMC10248468; doi:10.3389/fmicb.2023.1198945)
Supplement: Supplementary file 1 [file Data_Sheet_1.pdf]

Supplementary Material

Translocation of outer membrane vesicles from enterohemorrhagic *Escherichia coli* O157 across the intestinal epithelial barrier

Daniel Krsek[1], Daniel Alejandro Yara[2], Hana Hrbáčková[1], Ondřej Daniel[1], Andrea Mančíková[1], Stephanie Schüller[2], Martina Bielaszewska[1*]

*** Correspondence:** Martina Bielaszewska: martina.bielaszewska@szu.cz

1 Supplementary Figures and Tables

1.1 Supplementary Figures

Supplementary Figure 1. Translocation of DiO-labeled OMVs from a non-pathogenic *E. coli* strain across polarized Caco-2 monolayers. DiO-labeled OMVs (lacking all EHEC O157 toxins, O157 LPS, and H7 flagellin) were applied into apical compartments of Transwell inserts with polarized Caco-2 monolayers. DiO fluorescence was measured in basolateral compartments (BC) after indicated time periods and expressed as percentage of the original OMV inoculum. Data are means ± standard deviations from three independent experiments.

Supplementary Figure 2. Densitometric quantification of immunoblot signals of virulence factors in OMVs isolated from basolateral compartments (BC OMVs) shown in Figure 1D. The signals of virulence factors in BC OMVs were normalized to OmpA signals and expressed as percentages of virulence factor signals in OMVs applied into apical compartments (AC OMVs).

Supplementary Figure 3. Dextran sulfate sodium (DSS) treatment disrupts tight junctions. Polarized Caco-2 monolayers were treated for 24 h with 3% DSS or remained untreated (no DSS). Zonula occludens-1 (ZO-1) **(A)** and occludin **(B)** were stained with anti-ZO-1 and anti-occludin mouse monoclonal antibody, respectively, and Alexa Fluor 488-conjugated goat anti-mouse IgG. Nuclei were stained with DAPI. Preparations were analyzed with a confocal laser-scanning microscope Leica TCS SP8 equipped with a 63x/1.4 immersion oil objective. Scale bars are 10 µm. Images are representative of three independent experiments.

Supplementary Figure 4. Effects of EHEC O157 OMVs on the cell viability and TJ morphology after 4 h of incubation of polarized Caco-2 monolayers with EHEC O157 OMVs. (**A**) Cell viability was determined by MTT assay. Cell culture medium was a negative control and Triton X-100 a positive control. Data are means ± standard deviations from three independent experiments.

(B) Confocal microscopy of TJ protein ZO-1. ZO-1 was stained with anti-ZO-1 antibody and Alexa Fluor 488-conjugated goat anti-mouse IgG. Nuclei were stained with DAPI. Preparations were analyzed with a confocal laser-scanning microscope Leica TCS SP8 equipped with a 63x/1.4 immersion oil objective. Cell culture medium was a negative control. Scale bars are 10 µm. Images are representative of two independent experiments.

Supplementary Figure 5. Confocal microscopy analysis of OMV translocation pathways, time points 15 min **(A-C)** and 40 min **(D, E)**. Polarized Caco-2 monolayers were incubated with EHEC O157 OMVs for the time indicated and stained for OMVs (anti-*E. coli* O157 LPS antibody and Cy3-conjugated goat anti-rabbit IgG), ZO-1 (anti-ZO-1 antibody and Alexa Fluor 488-conjugated goat anti-mouse IgG), and nuclei (DAPI). Preparations were analyzed with a confocal laser-scanning microscope Leica TCS SP8 with a 63x/1.4 immersion oil objective. 3D images were acquired with Leica LAS X 3D viewer. **(A)** Side view of 3D image demonstrating localization of OMVs within ZO-1 after 15 min. **(B)** Localization of OMVs inside cells and between cells in colocalization with ZO-1 (yellow signals depicted by arrow heads). The main panel shows merged XY images and the side panels orthogonal XZ and ZY projections. **(C)** XY image of the region shown in panel (B) at the level of nuclei. **(D)** Side view of 3D image demonstrating localization of OMVs below ZO-1 and around nuclei after 40 min. **(E)** Merged XY image showing localization of OMVs inside cells. Scale bars are 10 µm. Images are representative of three independent experiments.

1.2 Supplementary Tables

Supplementary Table 1. Characteristics of *E. coli* O157 strains and OMVs used in this study

OMVs from strain	Serotype	Stx2a	CdtV	EHEC-Hly	H7 flagellin	O157 LPS (ng/ml)	OMV diameter (nm)	OMV counts (particles/ml x 10^{10})	OMV protein conc. (µg/ml)
5791/99	O157:H7	+	+	+	+	879±71	149.4±56.8	2.1±0.9	448±53
258/98-1	O157:H^-[H7]	+	+	-	-	867±81	148.9±37.8	1.9±0.8	427±64
258/98-2	O157:H^-[H7]	-	-	-	-	854±78	152.7±32.9	1.6±0.9	414±45
85-170	O157:H7	-	ND	ND	ND	ND	167.0±27.2	ND	541±14

Legend:

H^-[H7] designates nonmotile strains which carry the *fliC*$_{H7}$ gene.

Presence of Shiga toxin 2a (Stx2a), cytolethal distending toxin V (CdtV), EHEC hemolysin (EHEC-Hly) and H7 flagellin in OMVs was determined by immunoblot (Bielaszewska et al., 2017). O157 lipopolysaccharide (LPS) was quantified with the LAL Chromogenic Endotoxin Quantitation Kit. OMV sizes and counts were determined by nanoparticle tracking analysis (Bauwens et al., 2017b), and protein concentrations with Roti-Nanoquant reagent. The values for OMV sizes, counts, LPS and protein concentrations are means ± standard deviations from three independent measurements. +, virulence factor present; -, virulence factor absent; ND = not determined.
