# Supplementary material for: Translocation of outer membrane vesicles from enterohemorrhagic Escherichia coli O157 across the intestinal epithelial barrier
Source: Front Microbiol. 2023 May 25;14:1198945. doi: 10.3389/fmicb.2023.1198945 (PMC10248468; doi:10.3389/fmicb.2023.1198945)
Supplement: Supplementary file 2 [file Data_Sheet_2.PDF]

**Supplementary Table S1.** Characteristics of *E. coli* O157 strains and OMVs used in this study

| OMVs from strain | Serotype | Stx2a | CdtV | EHEC-Hly | H7 flagellin | O157 LPS (ng/ml) | OMV diameter (nm) | OMV counts (particles/ml x $10^{10}$) | OMV protein conc. (µg/ml) |
|---|---|---|---|---|---|---|---|---|---|
| 5791/99 | O157:H7 | + | + | + | + | 879±71 | 149.4±56.8 | 2.1±0.9 | 448±53 |
| 258/98-1 | O157:$H^-$[H7] | + | + | - | - | 867±81 | 148.9±37.8 | 1.9±0.8 | 427±64 |
| 258/98-2 | O157:$H^-$[H7] | - | - | - | - | 854±78 | 152.7±32.9 | 1.6±0.9 | 414±45 |
| 85-170 | O157:H7 | - | ND | ND | ND | ND | 167.0±27.2 | ND | 541±14 |

Legend:

$H^-$[H7] designates nonmotile strains which carry the *fliC*$_{H7}$ gene.

Presence of Shiga toxin 2a (Stx2a), cytolethal distending toxin V (CdtV), EHEC hemolysin (EHEC-Hly) and H7 flagellin in OMVs was determined by immunoblot (Bielaszewska et al., 2017). O157 lipopolysaccharide (LPS) was quantified with the LAL Chromogenic Endotoxin Quantitation Kit. OMV sizes and counts were determined by nanoparticle tracking analysis (Bauwens et al., 2017b), and protein concentrations with Roti-Nanoquant reagent. The values for OMV sizes, counts, LPS and protein concentrations are means ± standard deviations from three independent measurements. +, virulence factor present; -, virulence factor absent; ND = not determined.
