# Supplementary figures and images for: Translocation of outer membrane vesicles from enterohemorrhagic Escherichia coli O157 across the intestinal epithelial barrier
Source: Front Microbiol. 2023 May 25;14:1198945. doi: 10.3389/fmicb.2023.1198945 (PMC10248468; doi:10.3389/fmicb.2023.1198945)

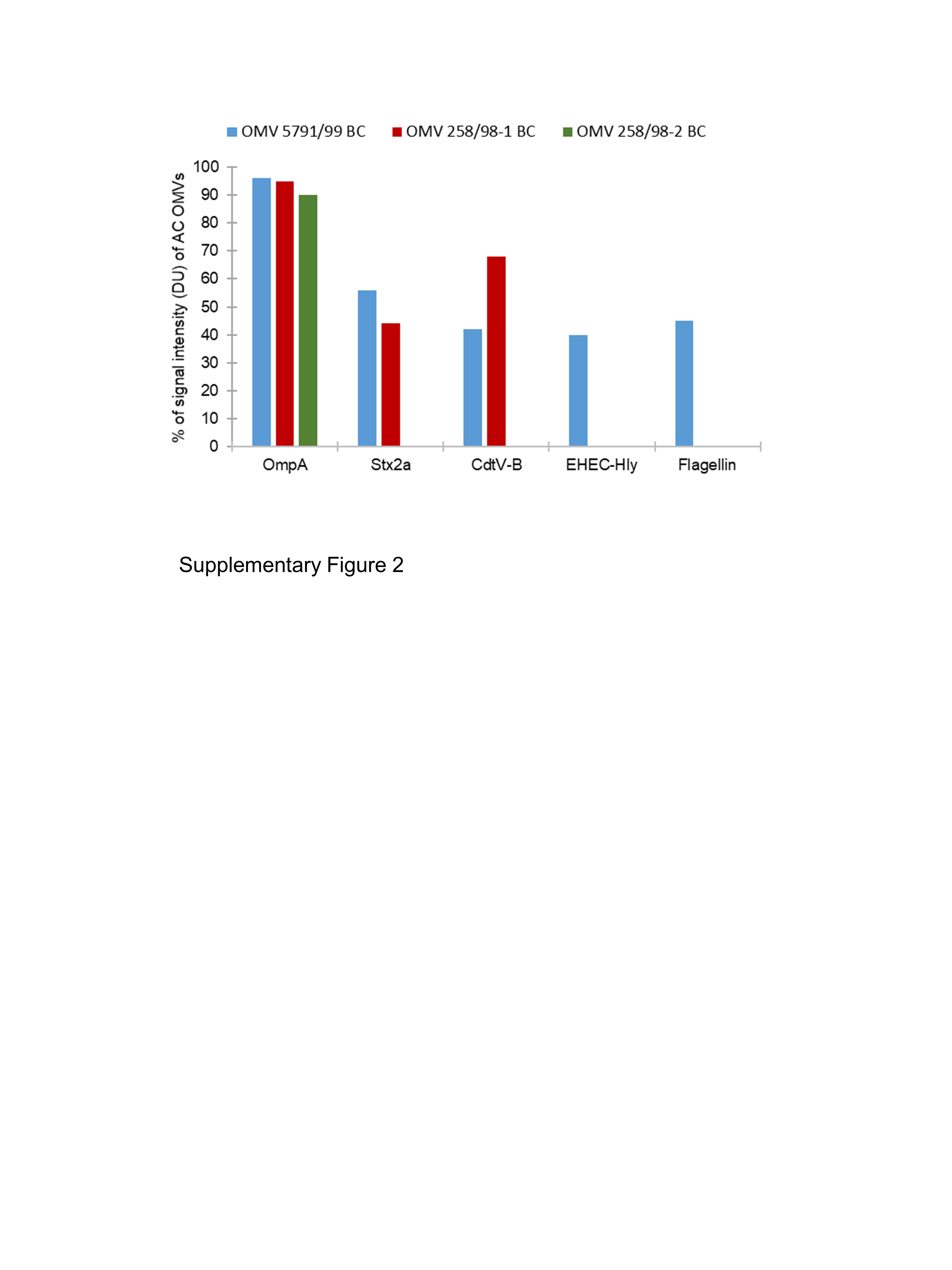

Supplement: Supplementary file 4 [file Image_2.tif]

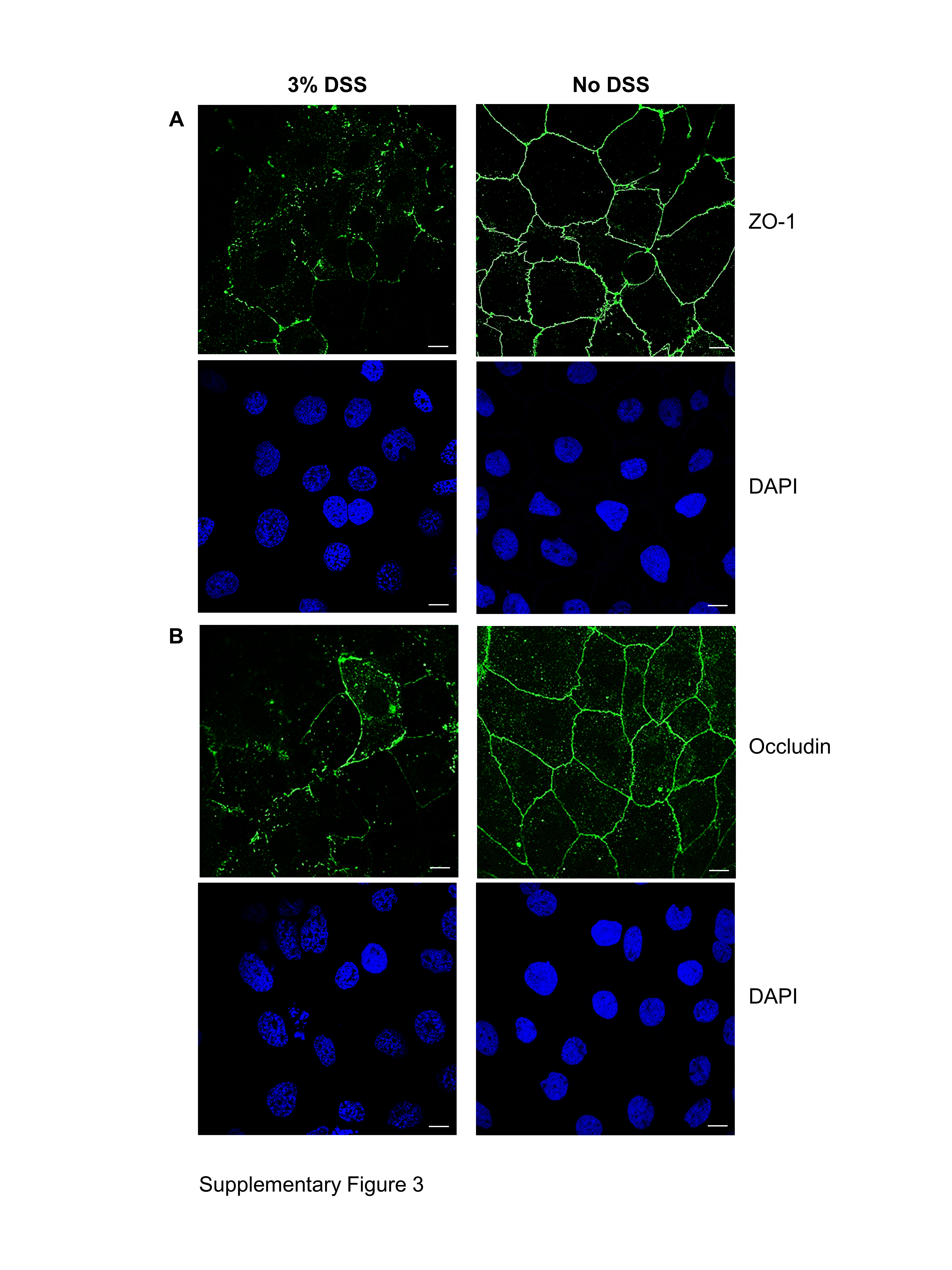

Supplement: Supplementary file 5 [file Image_3.tif]

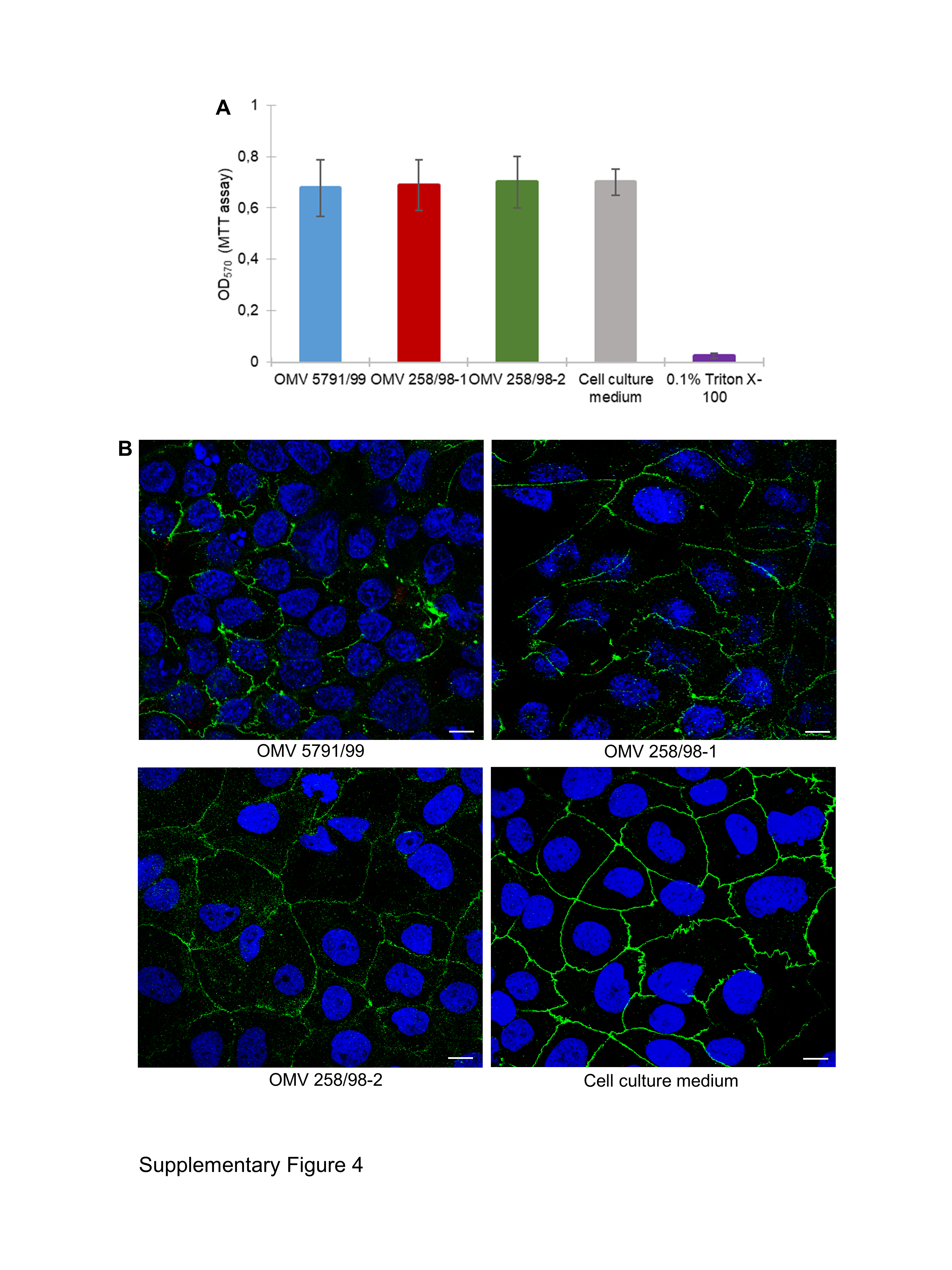

Supplement: Supplementary file 6 [file Image_4.tif]

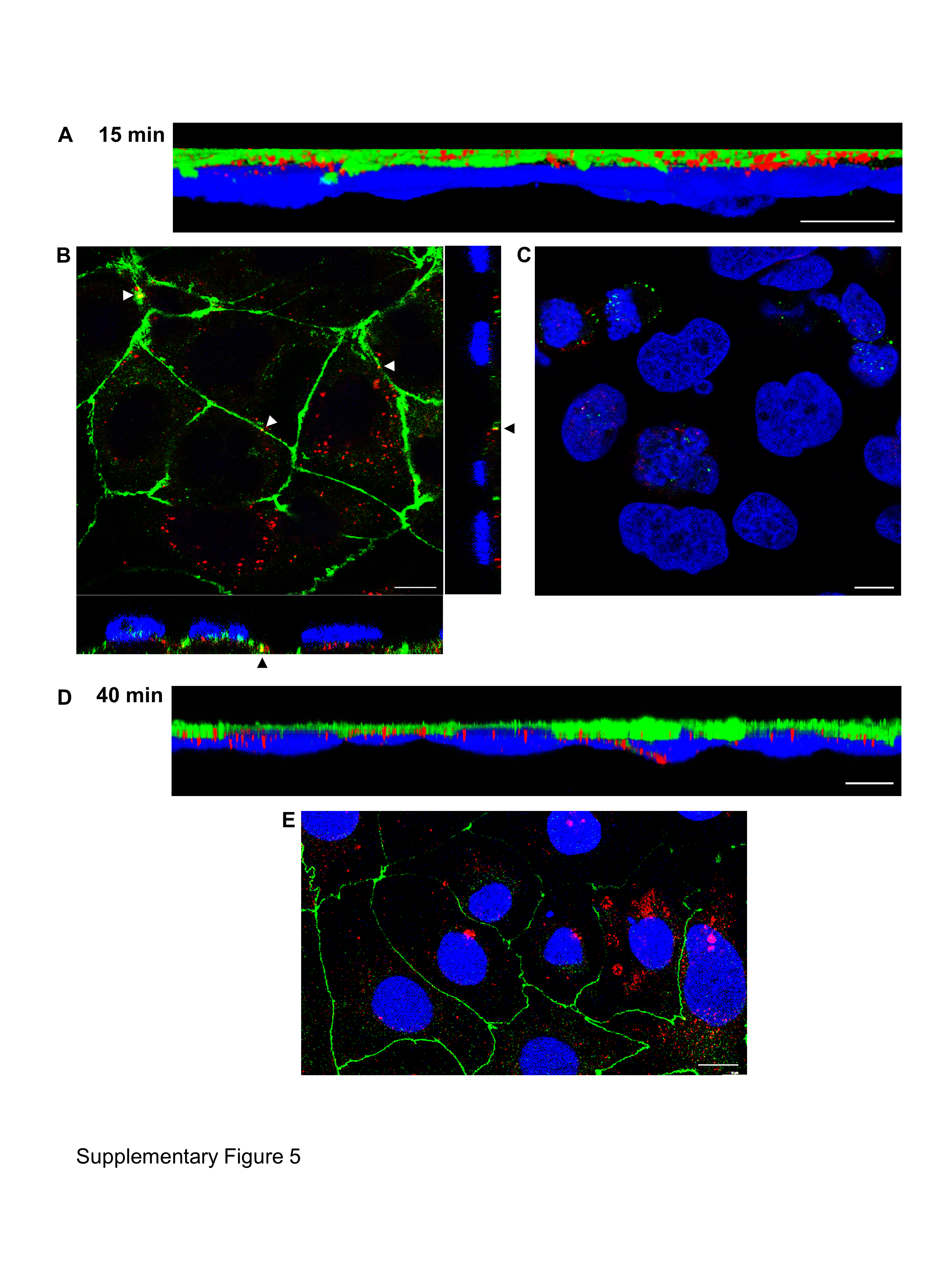

Supplement: Supplementary file 7 [file Image_5.tif]
